# Long-Term Flywheel Resistance Training Enhances Cognitive Flexibility in Older Women: A 36-Week Randomized Controlled Trial

**DOI:** 10.3390/jfmk11030325

**Published:** 2026-08-21

**Authors:** Maria Luiza da Cruz Santos, Pablo Augusto Garcia Agostinho, Wanderson Matheus Lopes Machado, Thalia Miranda Rufino, Leonardo Silveira Goulart Silva, Isabella Evangelista Leite, Cláudia Eliza Patrocínio de Oliveira, Édison Andrés Pérez-Bedoya, Osvaldo Costa Moreira

**Affiliations:** 1 Department of Physical Education, Federal University of Viçosa—Viçosa Campus, Viçosa 36570-900, Minas Gerais, Brazil; maria.l.cruz@ufv.br (M.L.d.C.S.); pablo.agostinho@ufv.br (P.A.G.A.); wanderson.machado@ufv.br (W.M.L.M.); thalia.rufino@ufv.br (T.M.R.); leonardo.goulart@ufv.br (L.S.G.S.); isabella.evangelista@ufv.br (I.E.L.); cpatrocinio@ufv.br (C.E.P.d.O.); 2 Human Morphophysiology Analysis Laboratory (HUMAN), Department of Physical Education, Federal University of Viçosa—Viçosa Campus, Viçosa 36570-900, Minas Gerais, Brazil; eaperez@elpoli.edu.co; 3 Faculty of Physical Education, Recreation and Sport, Politécnico Colombiano Jaime Isaza Cadavid, Medellín 050022, Antioquia, Colombia

**Keywords:** eccentric action, executive function, older women, female, strength training

## Abstract

**Background**: Executive functions (EFs) decline with aging, impacting functional independence. While resistance training (RT) is a promising non-pharmacological intervention, the optimal modality and duration for cognitive benefits remain unclear. Flywheel RT (FRT) imposes greater motor control and attentional demands than traditional RT (TRT), potentially enhancing EFs. **Objective**: To compare the effects of a 36-week FRT versus TRT program on executive function and serum insulin-like growth factor 1 (IGF-1) levels in healthy older women. **Methods**: This parallel-group randomized controlled trial allocated 64 older women (overall mean age 66.8 ± 4.6 years; FRT group: 68.0 ± 4.8 years; TRT group: 65.5 ± 4.1 years) to either FRT (n = 25) or TRT (n = 21) for two weekly sessions over 36 weeks. Executive function was assessed using the Victoria Stroop Test (inhibitory control), Digit Span Test (working memory), and Trail Making Test (cognitive flexibility). Serum IGF-1 was quantified via chemiluminescence. Intention-to-treat analyses with ANCOVA were performed. **Results:** After 36 weeks, both groups showed significant within-group improvements in inhibitory control (mean reduction from pre to post: FRT: 15.2 ± 10.4 to 14.8 ± 7.4 s; TRT: 20.4 ± 11.8 to 15.6 ± 7.3 s; *p* = 0.028, ηp^2^ = 0.38) and working memory (Digit Span Forward: FRT: 6.2 ± 1.7 to 7.9 ± 2.0; TRT: 6.7 ± 1.5 to 8.7 ± 1.8; *p* < 0.001, ηp^2^ = 0.84). The B/A ratio of the Trail Making Test improved significantly more in the FRT group (73.0 ± 65.2 to 87.5 ± 68.1 s) compared to the TRT group (67.3 ± 54.1 to 137.7 ± 83.6 s; group × time interaction: *p* = 0.001, ηp^2^ = 0.13). No significant changes in serum IGF-1 concentrations were observed in either group (FRT: 112.9 ± 25.4 to 115.3 ± 21.3 ng/mL; TRT: 104.7 ± 27.5 to 103.0 ± 28.9 ng/mL; *p* = 0.88, ηp^2^ = 0.002). Adherence was high (only 4 out of 46 participants did not complete all 72 sessions), and no severe adverse events were reported. **Conclusions**: Flywheel training appears particularly effective for enhancing cognitive flexibility, likely due to its higher cognitive-motor demands. The absence of significant changes in circulating IGF-1 suggests that the observed cognitive benefits may not be mediated by systemic endocrine pathways, although this does not exclude the possibility of local central nervous system adaptations or changes in other neurotrophic factors not assessed in this study.

## 1. Introduction

Accelerated population aging has emerged as a major challenge for healthcare systems and social organization [1]. The growing older population is likely to increase the number of individuals susceptible to cognitive decline and neurodegenerative diseases, including dementia, imposing a substantial social, economic, and healthcare burden that directly affects functional autonomy and the demand for care [2,3].

Among the cognitive domains most sensitive to physiological aging are executive functions (EFs), a set of higher-order cognitive abilities responsible for regulating goal-directed behavior [4,5]. EFs are understood through three core components: inhibitory control (IC), which suppresses automatic responses and inappropriate impulses; working memory (WM), which maintains and manipulates relevant information; and cognitive flexibility (CF), which enables the adaptation of cognitive and behavioral strategies in response to environmental changes [2,5,6]. Alterations in these executive components may impair the performance of activities of daily living, functional independence, and the ability to plan, monitor, and adapt behavioral responses [7,8]. Furthermore, poorer executive performance is associated with gait alterations and a greater vulnerability to falls in older adults [9].

Given the limitations of pharmacological approaches—including high cost, limited efficacy, and potential adverse effects—non-pharmacological interventions based on physical exercise have assumed a central role in preserving cognitive and functional health in older adults [10,11,12]. In this context, resistance training (RT) is widely recognized for attenuating musculoskeletal changes, preserving muscle strength, and helping prevent sarcopenia [13,14]. Recent studies have broadened this understanding by suggesting the potential of RT for brain health and the preservation of EFs [15,16,17]. These benefits have been associated with mechanisms such as functional neuroplasticity, increased cerebral perfusion and blood flow, and the modulation of neurotrophic and metabolic factors, including brain-derived neurotrophic factor (BDNF) and insulin-like growth factor 1 (IGF-1), both of which are involved in neural plasticity and brain metabolism [18,19].

In this context, resistance training (RT) is widely recognized for attenuating musculoskeletal changes, preserving muscle strength, and helping prevent sarcopenia [13,14]. Recent studies have broadened this understanding by suggesting its potential for brain health and the preservation of executive functions (EFs) [15,16,17]. These benefits have been associated with mechanisms such as functional neuroplasticity, increased cerebral perfusion and blood flow, and the modulation of neurotrophic and metabolic factors, including brain-derived neurotrophic factor (BDNF) and insulin-like growth factor 1 (IGF-1), both of which are involved in neural plasticity and brain metabolism [18,19].

Despite these promising findings, the literature on the effects of RT on EFs remains heterogeneous. Short-term randomized controlled trials (≤12 weeks) have often failed to detect significant improvements in certain executive domains, particularly cognitive flexibility [10,20,21], whereas longer interventions (≥6 months) appear to be more consistently beneficial [22]. Moreover, most studies have employed traditional resistance training modalities, leaving the potential of alternative RT approaches—especially those with greater cognitive-motor demands—largely unexplored. Factors such as protocol duration, motor complexity of the exercises, and the specificity of cognitive tests may influence the magnitude of cognitive changes in older adults [23,24,25].

Flywheel resistance training (FRT) emerges as a promising approach because it presents neuromechanical characteristics that differ from those of traditional resistance training (TRT) [15,26]. By using the inertia generated by a system composed of a shaft, cord, and rotating disc, FRT requires the kinetic energy produced during the concentric phase to be actively decelerated during the eccentric phase. This eccentric braking imposes greater demands on motor and executive control, coordination, postural adjustment, and attention during exercise [10]. Critically, the variable and unpredictable resistance inherent to the flywheel—which changes with each repetition depending on the applied acceleration—forces the practitioner to continuously adapt their motor strategy. This continuous motor re-adjustment is conceptually analogous to the cognitive processes required in flexibility tasks, which demand rapid reconfiguration of mental sets in response to changing environmental demands [4,5]. We posit that this ‘cognitive-motor dual-tasking’ may specifically engage and train the frontoparietal networks underlying cognitive flexibility [18]. However, there is still no robust body of evidence on the effects of FRT on EFs, and existing studies are based on short-term interventions (≤12 weeks). To the best of our knowledge, no long-term RCTs (≥36 weeks) have primarily focused on EFs in older women.

Given this gap, the primary aim of this study was to evaluate and compare the effects of 36 weeks of TRT and FRT on executive functions in older women. The secondary aims were to investigate the occurrence of adverse events, adaptations in muscle strength, and changes in serum IGF-1 concentrations. The central hypothesis was that both modalities would promote significant improvements in EFs; however, FRT would produce superior results, particularly in the cognitive flexibility component, due to the greater demands for motor control, attention, and braking imposed by isoinertial training. The relevance of this study lies in demonstrating, for the first time in a long-term RCT, that FRT is particularly effective in improving cognitive flexibility in older women and that the benefits in executive functions may occur independently of changes in IGF-1, suggesting direct neural mechanisms.

## 2. Materials and Methods

### 2.1. Study Design

This was a two-arm, parallel-group randomized controlled trial. The protocol followed the SPIRIT 2013 recommendations for the development of clinical trial protocols and the CONSORT guidelines for non-pharmacological interventions. The methodological quality of this RCT was assessed using the TESTEX tool [27].

### 2.2. Outcomes

Sociodemographic information, chronic diseases, comorbidities, vital signs, waist circumference, long-term medication use, time spent in sedentary behavior, and the number of falls in recent months were recorded at baseline using researcher-developed questionnaires.

All baseline sociodemographic and clinical information was collected using paper-based, structured questionnaires developed by the research team. The questionnaires were printed on standardized forms and administered in a quiet, private room at the Human Morphophysiological Analysis Laboratory (HUMAN LAB) to ensure participant comfort and confidentiality. After completion, the forms were manually checked for completeness by the research assistant and subsequently stored in a locked filing cabinet, with data later double-entered into an electronic spreadsheet (Microsoft Excel) for verification and analysis.

The questionnaires were administered in a mixed format: (a) sociodemographic questions (age, education level, marital status, occupation, retirement status) and lifestyle information (sedentary behavior, physical activity level, history of falls) were self-administered by the participants, with the research assistant available to clarify any doubts regarding the wording of the questions; (b) clinical and health-related information (chronic diseases, comorbidities, medication use, vital signs, waist circumference, and contraindications to exercise) were collected through an interviewer-administered format, conducted by trained research assistants (physical education students and postgraduate students) who read the questions aloud and recorded the participants’ responses. The interviewer-administered format was chosen for clinical questions to ensure accurate interpretation and to minimize missing data, particularly for participants with lower literacy levels.

All questionnaires were completed during the baseline assessment visit, which occurred before randomization and before the two-week familiarization period. Specifically, the questionnaires were administered on the same day as the initial in-person eligibility assessment, immediately after participants signed the informed consent form and before any physical measurements or cognitive tests were performed. This sequence was adopted to ensure that participants’ responses were not influenced by prior knowledge of their group allocation or by fatigue from the subsequent assessments. The baseline assessment visit took place approximately two to three weeks before the start of the intervention, allowing sufficient time for data entry, verification, and randomization procedures.

The primary outcome of this study was executive function, whereas secondary outcomes included adverse events, muscle strength, and serum IGF-1 concentrations.

### 2.3. Sample Size Calculation

The sample size was estimated based on randomized controlled trial (RCT) that investigated the effects of resistance training on physical function, assessed using the Timed Up and Go (TUG) test, in older adults [28]. Effect sizes were interpreted as follows: 0.01 was considered a small effect, 0.06 a moderate effect, and 0.14 or greater a large and substantial effect. A significance level of 5% (α = 0.05), statistical power of 80% (1 − β = 0.80), and a 1:1 allocation ratio between groups were adopted, resulting in a minimum estimated sample size of 60 participants. To compensate for potential sample losses during follow-up, an additional attrition rate of 15% was considered. The calculation was performed using EPIDAT software, version 4.2.

### 2.4. Blinding, Registration and Ethics Aspects

The principal investigator and the outcome assessor were blinded. However, the older women and the training supervisors were not blinded. The intervention period lasted 36 weeks. A two-week familiarization period preceded the interventions. Outcomes were assessed before the familiarization period, at the end of the fifth month, and one week after completion of the training program. The study was approved by the Ethics Committee of the Universidade Federal de Viçosa (UFV) under approval number 1.821.139 and was registered as a clinical trial (NCT06758206).

All potential participants were invited to an information session held at the Human Morphophysiological Analysis Laboratory (HUMAN LAB) prior to the start of the study. During this session, the principal investigator and trained research assistants provided a detailed verbal and written explanation of the study’s objectives, design, duration (36 weeks), and all procedures involved, including: (a) the two weekly resistance training sessions; (b) the neuropsychological assessments (Victoria Stroop Test, Digit Span Test, and Trail Making Test); (c) blood sample collection for IGF-1 analysis; (d) the one-repetition maximum (1RM) strength tests; and (e) the anthropometric and vital sign measurements. Participants were also informed about the schedule of assessments (pre-intervention, at 18 weeks, and post-intervention) and the expected time commitment for each visit.

The potential risks and discomforts associated with participation were thoroughly explained. These included: (a) transient musculoskeletal soreness or fatigue typically associated with resistance training; (b) the possibility of minor injuries (e.g., muscle strains or joint discomfort) during the training sessions, which would be promptly managed by the supervising physical education professionals; (c) the discomfort of venipuncture for blood collection; and (d) the mental effort required during cognitive testing. Participants were assured that all training sessions would be closely supervised to minimize risks, and that any adverse events would be documented and managed according to established protocols. The potential benefits were also clearly presented, including possible improvements in muscle strength, physical function, and cognitive performance, as well as the contribution of their participation to scientific knowledge on healthy aging and exercise prescription.

All participants were explicitly informed that their participation was entirely voluntary and that they had the right to withdraw from the study at any time, for any reason, without any negative consequences or prejudice to their future care or relationship with the researchers or the university. They were also assured that all data collected would be kept confidential and used solely for research purposes, with anonymity guaranteed in any publications or presentations. Participants were informed that their personal information would be stored in a secure, password-protected database accessible only to the research team, in compliance with the Brazilian General Data Protection Law (LGPD—Lei Geral de Proteção de Dados).

After receiving all information and having the opportunity to ask questions and clarify any doubts, participants who agreed to take part were asked to sign a free and informed consent form (Termo de Consentimento Livre e Esclarecido—TCLE), in duplicate, with one copy retained by the participant and the other kept by the research team. The consent form was written in clear, accessible language, consistent with the literacy level of the target population, and was approved by the Institutional Research Ethics Committee of the Federal University of Viçosa (CAAE: 60303716.1.0000.5153; approval number 1.821.139; approval date: 31 March 2025). All procedures complied with the principles of the Declaration of Helsinki and Resolution 466/2012 of the Brazilian National Health Council.

### 2.5. Participants

Potential participants were recruited from the community-dwelling older adult population residing in the urban area of Viçosa, Minas Gerais, Brazil, and its surrounding districts. Recruitment strategies included: (1) announcements disseminated by the Viçosa Municipal Health Department through primary healthcare units (Unidades Básicas de Saúde—UBS) and community health agents; (2) advertisements broadcast on local radio stations; (3) distribution of printed flyers at senior community centers, churches, and public squares; and (4) email invitations sent to retired university staff and members of local senior associations. All interested individuals were instructed to contact the research team by telephone or email for initial screening and scheduling of the first assessment visit.

Eligibility screening was conducted in two phases. The initial telephone screening was performed by trained research assistants (physical education and postgraduate students) who were calibrated by the principal investigator. During this call, they collected preliminary information regarding age, sex, physical activity level (using a brief questionnaire based on the ACSM recommendations), history of neurological or psychiatric diseases, and participation in resistance training programs in the previous three months. Individuals who met the preliminary inclusion criteria were invited for an in-person assessment.

At the Human Morphophysiological Analysis Laboratory (HUMAN LAB), candidates underwent a comprehensive in-person evaluation that included: (a) verification of vital signs (resting heart rate and blood pressure); (b) measurement of anthropometric variables (body mass, height, and waist circumference); (c) a detailed medical history interview to identify any contraindications to physical exercise (e.g., uncontrolled hypertension, severe cardiovascular disease, recent surgery, or severe musculoskeletal disorders); and (d) confirmation of literacy and visual capacity to perform the neuropsychological tests. Candidates also completed a questionnaire on sociodemographic characteristics, comorbidities, medication use, and history of falls. The final confirmation of eligibility was performed by the principal investigator, a certified physical education professional with more than 10 years of experience in exercise prescription for older adults, in conjunction with a physician consulted on an as-needed basis for complex medical cases. All eligibility decisions were documented in a standardized screening log.

The inclusion criteria were women aged ≥60 years, literate, with normal or corrected-to-normal vision, performing <150 min of moderate-to-vigorous physical exercise per week, and who provided written informed consent. The exclusion criteria included contraindications to physical exercise, neurological diseases (e.g., Alzheimer’s disease, Parkinson’s disease, stroke, or other dementias), or participation in resistance training programs in the previous three months (Table 1). Randomization was performed in blocks of 2 and 4, using opaque envelopes for allocation concealment.

Participants were considered eligible if they were community-dwelling, functionally independent, and had no self-reported or medically diagnosed neurological diseases (e.g., Alzheimer’s disease, Parkinson’s disease, stroke, or other dementias) or psychiatric disorders that could affect cognitive performance or adherence to the training program. Although participants were not systematically screened using a standardized cognitive battery (e.g., Mini-Mental State Examination—MMSE) prior to inclusion, none had a prior diagnosis of mild cognitive impairment or dementia, and all were capable of providing informed consent and understanding the test instructions. This is consistent with the pragmatic nature of the trial, which aimed to recruit generally healthy, community-dwelling older women without known cognitive pathology.

Physical activity level was assessed using a standardized questionnaire developed by the researchers, based on the recommendations of the American College of Sports Medicine (ACSM). Participants were asked to report the frequency, duration, and type of physical activities performed in a typical week over the previous three months. Only those who reported engaging in less than 150 min of moderate-to-vigorous physical activity per week—and who had not participated in any structured resistance training program in the previous three months—were included. This threshold was chosen to ensure that participants were not regularly physically active, thereby minimizing the potential confounding effects of pre-existing exercise habits on the outcomes.

The random allocation sequence was generated by an independent researcher not involved in the recruitment, eligibility assessment, or intervention delivery (E.A.P.B.), using a computer-generated random number table (Microsoft Excel, version 2023) with a 1:1 allocation ratio between the two groups (FRT and TRT). Randomization was performed in permuted blocks of sizes 2 and 4, randomly varied to ensure concealment of the block size and to prevent predictability of group assignment. The block sequence was generated prior to the start of recruitment and was not disclosed to any member of the research team involved in participant assessment or training supervision.

The sealed opaque envelopes were prepared by the same independent researcher (E.A.P.B.) who generated the allocation sequence. Each envelope was sequentially numbered from 1 to 64 and contained a card indicating the group assignment (FRT or TRT). The envelopes were identical in appearance (opaque, sealed, and of the same color and size) to ensure that the sequence remained concealed. They were stored in a locked cabinet in a secure office, accessible only to the independent researcher, and were not opened until the moment of randomization.

**Figure 1:**
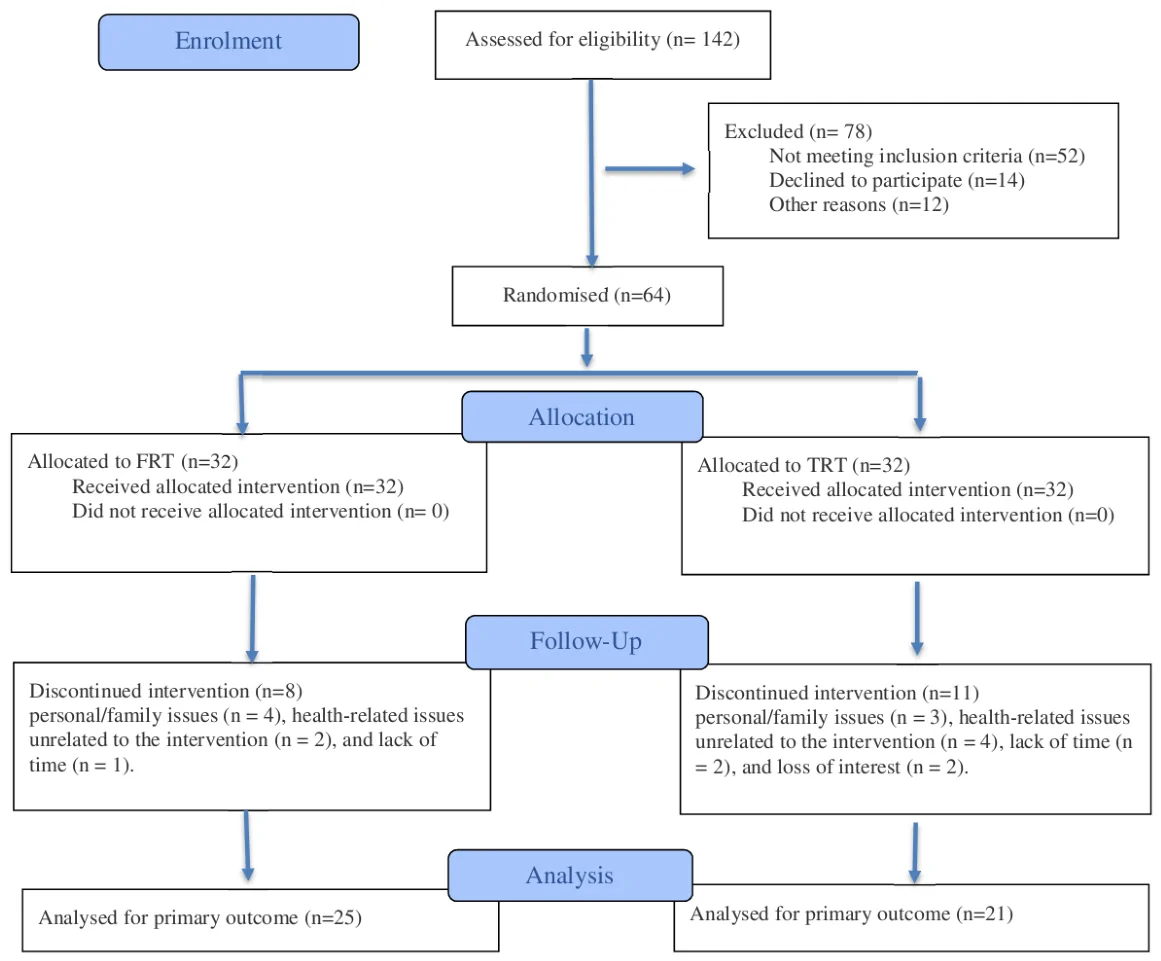
CONSORT 2025 Flow Diagram.

After completing all baseline assessments and confirming eligibility, each participant was assigned to the next sequentially numbered envelope by a research assistant (M.L.C.S.) who was not involved in the generation of the allocation sequence and had no knowledge of the block sizes or the sequence order. The research assistant opened the envelope in the presence of the participant and recorded the group assignment in a secure, password-protected log. The participant was then scheduled for the familiarization sessions according to their allocated group. This procedure ensured that the allocation was concealed from both the outcome assessor and the principal investigator until the moment of assignment. The participant flow through enrollment, allocation, follow-up, and analysis is presented in Figure 1.

The following measures were implemented to minimize allocation bias: (a) the random allocation sequence was generated by an independent researcher (E.A.P.B.) who was not involved in participant recruitment, eligibility screening, outcome assessment, or intervention delivery; (b) the sealed envelopes were prepared by the same independent researcher, ensuring that no member of the research team with direct participant contact had prior knowledge of the sequence; (c) group assignment was performed by a research assistant (M.L.C.S.) who was trained in the randomization protocol but was not involved in generating the sequence or preparing the envelopes; and (d) the outcome assessor (P.A.G.A.) and the principal investigator (O.C.M.) were blinded to group allocation until after the completion of all post-intervention assessments and data analysis, as described in the Blinding section. Thus, the randomization and allocation procedures were conducted by personnel independent of the recruitment and assessment processes, ensuring adequate concealment and minimizing the risk of allocation bias.

### 2.6. Resistance Training Program

The resistance training interventions were described using the Consensus on Exercise Reporting Template (CERT) [29]. The CERT provides a standardized framework for describing exercise interventions, ensuring that key elements such as exercise type, intensity, duration, frequency, and progression are reported consistently and accurately.

Training sessions were conducted on weekdays (Monday to Friday), with each participant attending two non-consecutive days per week (with at least 48 h of rest between sessions). Sessions were scheduled between 7:00 AM and 6:00 PM. Within this window, individual start times were fixed for each participant throughout the 36-week intervention (e.g., a participant who started at 8:30 AM continued at the same time for all subsequent sessions). This standardization was implemented to minimize the potential confounding effects of diurnal variations in performance, motivation, and physiological responses.

The training schedule was standardized across participants regarding: (a) the total number of sessions per week (two); (b) the minimum rest interval between sessions (48 h); (c) the time of day; (d) the order of exercises (always starting with lower-body exercises followed by upper-body exercises); (e) the number of sets and repetitions (four sets of eight coupled concentric and eccentric actions for FRT; four sets of eight to twelve repetitions for TRT); and (f) the rest intervals between sets (120 s for FRT; 60 s for TRT). However, the exact start time was allowed to vary slightly between participants to accommodate individual availability and to avoid overcrowding in the training facilities. Each participant’s individual start time remained consistent across all sessions throughout the study. Supervision was provided individually or in groups of up to three participants, ensuring that all participants received equal attention and instruction regardless of their scheduled time.

Participants in the flywheel resistance training group (FRT) used a multi-leg isoinertial device (Physical Solutions, São Paulo, Brazil) located at the Human Morphophysiological Analysis Laboratory (HUMAN LAB), whereas participants in the traditional resistance training group (TRT) used weight machines and free weights at the university gym of the Department of Physical Education. All interventions were supervised by Physical Education professionals or students with previous experience in strength training and trained for the study, and were conducted individually or in groups of up to three participants.

Adherence was monitored through the perceived exertion reported by the participants at the end of each session. To maintain motivation and encourage continued participation among the older women, regular discussions were held to provide information about their progress. Participants also received guidance and feedback on their performance and improvement in exercise execution.

Each session consisted of six general exercises—knee extension and flexion, plantar flexion, scapular retraction, elbow flexion, and shoulder flexion—standardized for both groups. Both groups performed two training sessions per week for 10 months, with 48 h of rest between sessions. The FRT performed four sets of eight coupled concentric and eccentric actions at high intensity, corresponding to a score of 10 on the OMNI-RES scale, with 120 s of rest between sets. Load progression was achieved by manipulating the speed used to move the flywheel, with an inertial load of 0.05 kg·m^2^, always at maximal concentric power and with braking during the final third of the movement. Load progression in FRT was autoregulated by speed and maximal concentric power, since the inertial load is fixed, but the effective resistance is determined by the acceleration applied to the flywheel. The TRT performed four sets of eight to 12 repetitions at moderate-to-high intensity, corresponding to scores of 6 to 10 on the OMNI-RES scale, with 60 s of rest. Load progression occurred when the participant completed more than 12 repetitions with proper execution and a time under tension of one second for the concentric phase and two seconds for the eccentric phase. No home-based exercise or non-resistance training component was included in the prescription. All adverse events were documented and managed.

### 2.7. Assessment Measures

#### 2.7.1. Executive Function

Executive function was assessed using three standardized neuropsychological tests selected to capture the main components of executive functions according to the models proposed by Miyake et al. [4] and Diamond [5]. All tests were administered by a trained assessor blinded to group allocation, in a quiet environment with adequate lighting.

Inhibitory control was assessed using the Victoria Stroop Test [30]. The test consists of three cards with 24 items each: (1) naming colors of colored dots, (2) naming the ink color of colored words in the neutral condition, and (3) naming the ink color of words that name different colors in the interference condition. The final score is the time, in seconds, required to complete the interference card, with shorter times indicating better inhibitory control.

Working memory was assessed using the Digit Span Forward and Backward Test [31]. In the Forward test, the examiner reads sequences of digits in increasing length, and the participant must repeat them in the same order. In the Backward test, the participant must repeat the sequence in reverse order. The final score is the total number of sequences correctly repeated in each condition, with higher scores indicating better capacity to retain and manipulate information.

Cognitive flexibility was assessed using the Trail Making Test Parts A and B [32,33]. In Trail Making Test Part A, the participant must connect numbers from 1 to 25, randomly distributed on a sheet of paper, in ascending order. In Trail Making Test Part B, the participant must alternate between numbers and letters in ascending order (1-A-2-B-3-C…). The final score is the time, in seconds, required to complete each part, with shorter times indicating better performance. The B/A ratio, calculated as the Trail Making Test Part B time divided by the Trail Making Test Part A time, was used as an additional indicator of cognitive flexibility, controlling for motor processing speed.

#### 2.7.2. Insulin-like Growth Factor 1 (IGF-1)

To measure IGF-1 concentration (ng/mL), blood samples were collected at Viçosa Lab, located in the city of Viçosa, Minas Gerais. Samples were collected after a 2-h fasting period. IGF-1 quantification was performed using chemiluminescence, a specific technique capable of detecting the substance of interest at extremely low levels with high precision.

#### 2.7.3. Adverse Events

Training-related events were categorized according to severity using the Common Terminology Criteria for Adverse Events (CTCAE v5.0), grades 1–5. Incidence was reported by group, with attribution recorded. Dropouts due to medical reasons were classified as grade 3 adverse events (AEs); grades 1 and 2 represented typical physiological responses to exercise and were recorded but were not considered preventable.

#### 2.7.4. Muscular Strength Assessment

Maximal dynamic strength was determined using the one-repetition maximum (1RM) test in the knee extension exercise. Participants remained seated, with their backs supported against the equipment backrest and knees flexed at 90°. The movement consisted of knee extension to approximately 180°, followed by a return to the initial position. Before the test, a warm-up was performed with four repetitions at 30% of maximal voluntary isometric contraction. The load was progressively increased according to execution and perceived exertion assessed using the OMNI-RES scale (0–10). A maximum of five attempts were performed, with a two-minute interval between attempts, and the load corresponding to one repetition maximum was recorded [34].

### 2.8. Statistical Analysis

The normality of the distribution of quantitative variables was assessed using the Shapiro–Wilk test, and Levene’s test was used to examine the homogeneity of variance. Normally distributed variables were presented as means and standard deviations. To compare baseline results between groups, Student’s t-test for independent samples was applied. Statistical differences were confirmed assuming equal variances and were further supported by analysis of the 95% confidence interval.

To assess intra- and intergroup differences, analysis of covariance (ANCOVA) was performed on the post-intervention (36-week) outcomes, with the baseline scores of the respective outcome variable included as a covariate. This approach was chosen because our primary research question focused on comparing the two groups at the end of the 36-week intervention period, while controlling for baseline performance. ANCOVA provides adjusted post-intervention means and is particularly appropriate when baseline differences between groups exist as observed for waist circumference and BMI (Table 2), increasing statistical power and reducing the risk of Type I error.

For each outcome, the following covariates were included: (a) for the Victoria Stroop Test, the baseline Stroop score; (b) for the Digit Span Forward test, the baseline Digit Span Forward score; (c) for the Trail Making Test B–A ratio, the baseline TMT B–A ratio; and (d) for IGF-1, the baseline IGF-1 concentration. Additional covariates such as age and years of education were considered but were not included in the final models, as preliminary analyses showed that they did not significantly improve the model fit (*p* > 0.05) or alter the direction or significance of the main effects.

The intermediate (18-week) assessment was analyzed descriptively and used to explore the trajectory of changes. Within-group changes from baseline to 18 weeks and from 18 weeks to 36 weeks were calculated. As a sensitivity analysis, we also performed a repeated-measures ANOVA including all three time points (baseline, 18 weeks, and 36 weeks) to confirm the robustness of our findings. The results of this sensitivity analysis were consistent with those obtained from the ANCOVA, with a significant time effect for inhibitory control and working memory, and a significant group × time interaction for cognitive flexibility.

Effect size was estimated using partial eta squared (ηp^2^), interpreted as small (0.01), moderate (0.06), or large (≥0.14). Statistical power was set at 80% (1 − β= 0.80). Within-group changes from baseline were calculated as the difference between final and initial values of the variables. The statistical significance level adopted for the analysis was α = 5%. Data analysis was performed using SPSS version 25 for Windows.

## 3. Results

A total of 142 older women were initially assessed for eligibility. Of these, 78 were excluded prior to randomization for the following reasons: not meeting the inclusion criteria (n = 52), declining to participate (n = 14), having contraindications to exercise (n = 7), or being unavailable for the full 36-week intervention period (n = 5). The remaining 64 participants were randomized into the two study groups. During the intervention period, an additional 18 participants discontinued the study (TRT: n = 11; FRT: n = 8), resulting in a final sample of 46 participants who completed the full 36-week protocol (TRT: n = 21; FRT: n = 25). Reasons for dropout included personal/family issues (n = 7), health-related issues unrelated to the intervention (n = 6), lack of time (n = 3), and loss of interest (n = 2).

The sample consisted of 25 White women (44.4%), 16 mixed-race/brown women (34.8%), and five Black women (10.9%). Among them, nine women (19.6%) had completed elementary school I, 15 women (32.6%) had completed elementary school II, 10 women (27.1%) had completed high school, and 12 women (26.1%) had completed higher education. Of these women, 36 were retired (78.3%) and 10 were not yet retired (21.7%); 33 performed some type of formal or informal work (71.7%), whereas 13 did not (28.3%). Regarding marital status, 17 women were married (37.0%), seven were widowed (15.2%), 11 were divorced (24.0%), and 11 were single (24.0%). The remaining sample characteristics for both groups are presented in Table 2.

After 36 weeks of training, it was found that, in the TRT, three of the 21 women did not complete the 72 planned training sessions due to personal and family issues. In the FRT, only one of the 25 women did not complete all sessions due to health-related issues.

Regarding the 1RM test, the mean value in the TRT group was 42.52 ± 12.79 kg at pre-intervention, 52.38 ± 8.61 kg at the mid-point, and 49.52 ± 11.72 kg post-intervention. In the FRT, the corresponding values were 39.60 ± 13.01 kg at pre-intervention, 51.24 ± 14.96 kg at the mid-point, and 51.80 ± 16.00 kg after the intervention. Thus, a significant within-group difference was observed after the intervention (*p* = 0.001); however, no significant between-group difference was found (*p* = 0.317).

**Figure 2:**
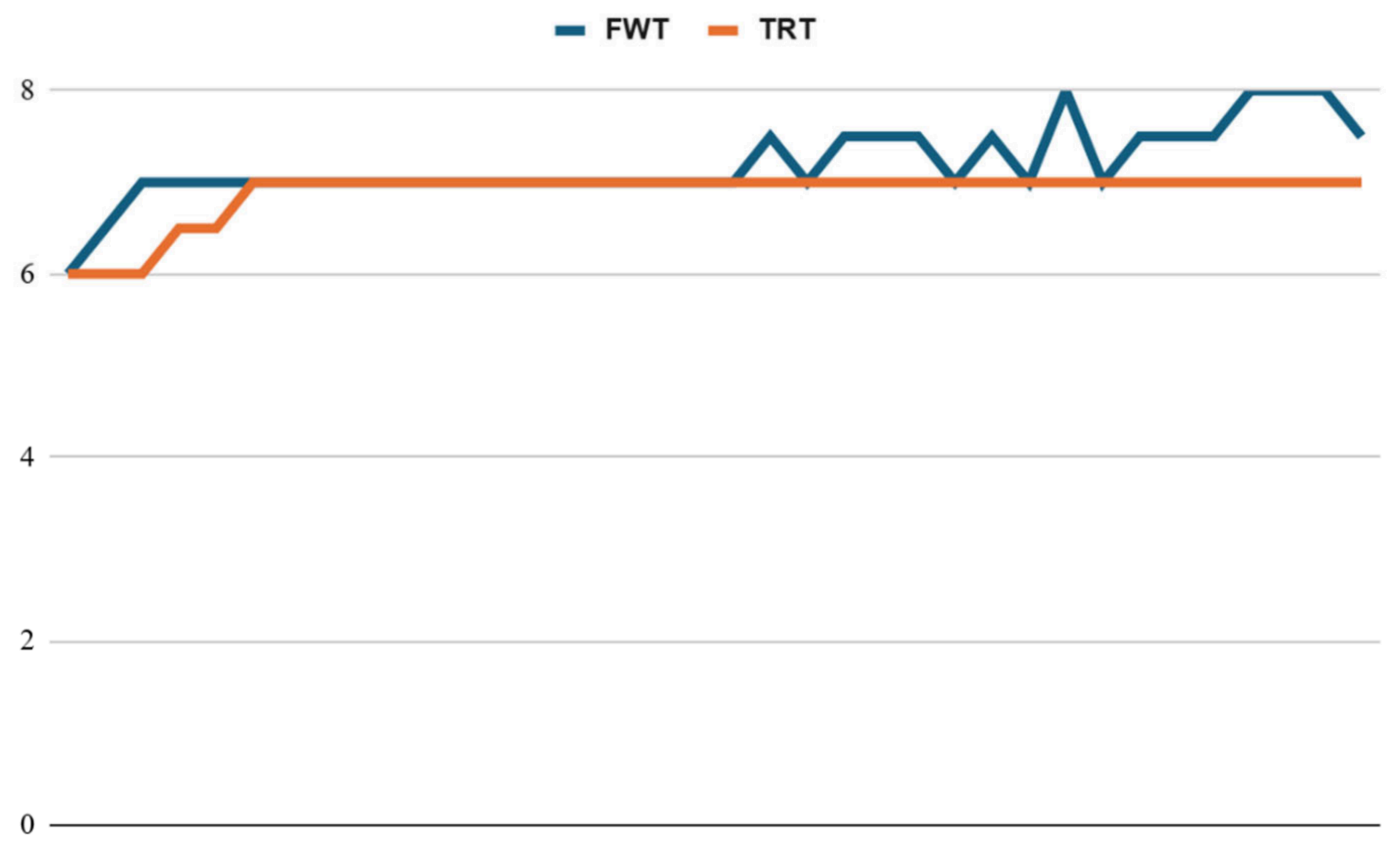
Mean perceived exertion response in each intervention group over the 36 weeks.

Figure 2 presents the mean perceived exertion responses in both groups over the 36 weeks. It indicates that both groups maintained a perceived exertion level classified as “somewhat hard” or “hard” according to the OMNI-RES scale.

Regarding adverse events (AEs), a total of 526 events were recorded in the FRT and 134 in the TRT. Most AEs were classified as grade 1, and no grade 3 to 5 AEs were observed. In the FRT, 233 AEs were considered training-related, mainly involving transient musculoskeletal symptoms. In the TRT, 18 AEs were related to training, and seven grade 2 AEs were recorded; however, these were not related to the intervention. Overall, the AEs did not result in functional limitations or interruption of the participants’ daily activities.

Table 3 presents the effects of the resistance training interventions on executive function indicators and IGF-1 levels in the volunteers. For the Victoria Stroop Test (inhibitory control), a reduction in scores was observed in both groups (*p* = 0.028, ηp^2^ = 0.38; 1 − β = 0.686), with the TRT showing superior performance compared with the FRT at the second assessment time point (*p* = 0.015, ηp^2^ = 0.23; 1 − β = 0.754). Regarding the Digit Span Forward test (working memory), significant differences were observed in both groups (*p* < 0.001, ηp^2^ = 0.84; 1 − β = 1.000). In the Trail Making Tests A and B, there was a group-by-time difference (*p* = 0.007, ηp^2^ = 0.486; 1 − β = 0.869), with the FRT showing a significantly greater improvement (*p* = 0.001). No significant difference was observed for IGF-1.

## 4. Discussion

To the best of our knowledge, this is the first longitudinal study to investigate the effects of flywheel resistance training (FRT) on executive functions in older women over a 36-week intervention period. Our main findings demonstrate that: (1) both types of resistance training, traditional and flywheel, improved inhibitory control and working memory; (2) both types improved cognitive flexibility, but with a significant superiority of the group submitted to FRT; (3) improvements in executive functions can already be observed after 18 weeks, regardless of the training modality; and (4) no significant changes in serum IGF-1 levels were observed over the 36-week intervention. These findings suggest that FRT may be a particularly effective strategy to mitigate the decline in cognitive flexibility in older women, possibly due to its greater cognitive-motor demand.

Both training protocols (FRT and TRT) promoted significant improvements in inhibitory control and working memory, suggesting that the RT stimulus, regardless of modality, is beneficial for these components of executive functions. Inhibitory control, which is essential for suppressing irrelevant stimuli and impulsive responses, allows individuals to maintain focus on relevant information, contributing to safer decision-making and the prevention of risk behaviors in older adults [5,27].

In agreement with our results, Liu-Ambrose et al. [22] demonstrated that 12 months of resistance training improved inhibitory control, assessed using the Stroop test, as well as selective attention and conflict resolution in older women. Complementarily, Eckardt et al. [20] observed that 10 weeks of training were sufficient to promote positive effects on the same component of executive functions. However, our data contrast with short-term studies, such as those by Cota et al. [10] and Iuliano et al. [21], which found no significant improvements in the Stroop test after 12 weeks of intervention. This divergence suggests that protocol duration may be a determining factor for the consolidation of resistance training-induced cognitive adaptations, and it is possible that 36 weeks represents the minimum time required for improvements in inhibitory control to manifest consistently in older women.

The improvement in inhibitory control observed in our study may be explained, at least in part, by neurofunctional adaptations in brain regions involved in executive control. Liu-Ambrose et al. [35] demonstrated that older women who underwent resistance training at least twice per week showed greater activity in the orbitofrontal cortex and left anterior insula, regions related to the inhibition of inappropriate responses, suggesting that resistance training may be associated with more efficient engagement of inhibitory processes.

Similarly, our findings for working memory are consistent with previous evidence indicating the positive effect of resistance training on this domain. Santos et al. [17] observed a significant improvement in working memory on the Digit Span test after 12 weeks of resistance training, and Cota et al. [10], when investigating FRT, also found a significant improvement in this component after 12 weeks. Specifically, for working memory, this effect may be related to greater involvement of the dorsolateral prefrontal cortex and frontoparietal networks, regions involved in learning, decision-making, and the maintenance of task-relevant information [5,36].

In contrast, Iuliano et al. [21] observed no significant improvements in working memory in a 12-week randomized controlled trial of resistance training, and Yoon et al. [37] also found no significant changes in Digit Span performance after 16 weeks. These contradictory findings highlight the heterogeneity of the literature and suggest that the benefits of resistance training for working memory may depend on factors such as intensity, weekly frequency, type of protocol, and, most importantly, intervention duration. Our 36-week data provide robust evidence that adaptations in working memory can be achieved with long-term resistance training in older women.

The most notable finding of our study was the superiority of FRT in improving cognitive flexibility, assessed using the Trail Making Test B–A. Cognitive flexibility, the ability to adapt to new situations and modify responses in response to environmental changes, is essential for functional independence, personal autonomy, and quality of life in older women [5,18]. Our data demonstrate that, although both protocols improved Trail Making Test performance, the group submitted to FRT showed a significantly greater magnitude of improvement (*p* = 0.001), suggesting a specific effect of this modality on this executive domain.

We believe this superiority is due to the greater demand for motor control and attention inherent to FRT. Unlike traditional resistance training, flywheel training requires the kinetic energy produced during the concentric phase to be actively decelerated during the eccentric phase, imposing greater demands on attention, postural control, continuous adjustment of force and velocity, and fine motor coordination [10,26]. The eccentric phase of FRT requires more complex motor planning and continuous force adjustment to brake the inertia, engaging the anterior cingulate cortex and frontoparietal networks, which are crucial regions for cognitive flexibility [10,18].

This interpretation is consistent with recent findings by Cota et al. [10], who observed that 12 weeks of FRT promoted a significant improvement in Trail Making Test performance, with a more pronounced improvement in the group submitted to FRT compared with TRT. Likewise, Eckardt et al. [20] observed that 10 weeks of resistance training on unstable surfaces, which also imposes greater motor-control demands, produced positive effects on cognitive flexibility. However, our study advances beyond these previous investigations by demonstrating that the effect of FRT is even more pronounced after 36 weeks of intervention, suggesting that the combination of greater motor complexity and longer training duration potentiates the benefits for cognitive flexibility.

Furthermore, it is important to consider the chronic neuromuscular adaptations promoted by resistance training, such as improved activation of fast-twitch fibers (type II) and greater efficiency in motor unit recruitment [3,38]. In FRT, the high eccentric demand appears to further potentiate neuromuscular recruitment and mechanical efficiency, allowing the same work to be performed with a lower relative need for motor unit recruitment and lower cortical activation during task execution [15]. Thus, movements that previously required high energetic and cognitive demands may become more automated and efficient throughout the training period, favoring better cognitive performance in tasks that require the prefrontal cortex [10,15].

Conversely, Cardoso et al. [39] observed no significant improvement in cognitive flexibility after 16 weeks of resistance training, and Forte et al. [40] also found no significant changes after 12 weeks. These findings suggest that the effects of resistance training on cognitive flexibility remain heterogeneous in the literature, possibly due to differences in protocol duration and methodological diversity across interventions. Our study suggests that 36 weeks represents a relevant methodological distinction, allowing longer exposure to resistance training and greater consolidation of the neuromuscular and neurofunctional adaptations associated with cognitive flexibility.

In our study, no significant pre- to post-intervention changes in serum IGF-1 levels were observed in either group. The absence of changes in IGF-1 contrasts with studies such as that by Parkhouse et al. [41], who observed a significant increase in IGF-1 after 32 weeks of resistance training in older women with low bone mineral density, but corroborates recent findings by Cota et al. [10] and Goekint et al. [42], who also found no significant changes despite cognitive improvements. This finding suggests that the cognitive benefits observed in our study may not be mediated by changes in circulating IGF-1 levels, and may instead involve alternative pathways, such as central neural mechanisms (e.g., functional neuroplasticity, increased cerebral perfusion, or synaptic efficiency) rather than systemic endocrine pathways [20,36]. However, it is important to note that we measured only serum IGF-1, and we cannot rule out the possibility of changes in local brain IGF-1 activity, central IGF-1 production, or other neurotrophic factors (e.g., BDNF, VEGF, IGFBP-3) that were not assessed in this study. Furthermore, serum IGF-1 concentrations do not necessarily reflect IGF-1 activity or bioavailability in the central nervous system, where autocrine and paracrine signaling within brain tissue may be critical for neural plasticity and cognitive function [18,19]. Therefore, our findings should be interpreted with caution, and future studies are needed to directly investigate central IGF-1 signaling and other potential mediators using neurophysiological and molecular approaches.

This interpretation is supported by evidence showing that resistance training may induce functional neuroplasticity through pathways that do not depend on peripheral trophic factors. Macaulay et al. [19] and Soshi et al. [43] suggest that neurovascular adaptations, increased neural efficiency, and greater engagement of cortical regions, particularly prefrontal areas associated with executive control, may occur independently of changes in circulating IGF-1 levels. We hypothesize that improvements in synaptic efficiency, hippocampal neurogenesis, or cerebral blood flow induced by exercise may underlie the cognitive effects observed.

Although IGF-1 is recognized as an important neurotrophic factor, with a documented role in neural plasticity and brain metabolism [18,19], the absence of changes in circulating IGF-1 in our study does not rule out the possibility of resistance training-induced neuroplasticity or the involvement of the IGF-1 system. First, circulating IGF-1 levels do not necessarily reflect central IGF-1 activity, as IGF-1 is produced both peripherally and centrally, and local autocrine and paracrine signaling within brain tissue may play a more critical role in cognitive function than systemic concentrations [18]. Second, changes in IGF-1 bioavailability, receptor sensitivity, or downstream signaling pathways (e.g., PI3K/Akt, MAPK/ERK) could occur without detectable changes in total serum IGF-1 levels. Third, other neurotrophic factors not assessed in this study, such as BDNF, VEGF, or IGFBP-3, may mediate the cognitive benefits of resistance training. Therefore, our findings do not exclude endocrine or neurotrophic involvement; rather, they suggest that the observed cognitive improvements may occur through mechanisms that are not reflected by changes in circulating IGF-1 concentrations. Future studies should incorporate a broader panel of biomarkers, including BDNF, VEGF, IGFBP-3, and inflammatory markers, as well as neurophysiological measures such as functional magnetic resonance imaging and electroencephalography, to more comprehensively characterize the biological pathways underlying the cognitive benefits of resistance training.

Although FRT generated a higher number of grade 1 musculoskeletal adverse events than TRT (233 vs. 18 training-related events), all were transient, did not result in functional limitations or interruption of the volunteers’ daily activities, and no grade 3 to 5 adverse events were observed. This suggests that FRT is a safe tool for older women, although it requires more careful familiarization and appropriate supervision during the initial phase of training, especially because of the greater eccentric demand and higher motor-control requirements of this modality. The high adherence to the protocol, with only four participants not completing the 72 planned sessions, reinforces the feasibility and acceptability of FRT in this population.

As a practical application, our results support the prescription of FRT as an effective non-pharmacological strategy to mitigate the decline in cognitive flexibility in older women. Physical education professionals and gerontologists may consider including FRT in long-term training programs for older adults, especially those at greater risk of cognitive decline or those requiring preservation of functional autonomy. The superiority of FRT over TRT for cognitive flexibility, combined with its safety and feasibility, makes this modality a promising tool for promoting brain health in the older population.

This study presents several notable strengths, including its longitudinal randomized controlled design with a 36-week intervention—substantially longer than most previous investigations—which allowed for the detection of robust and sustained cognitive and physical adaptations. The direct, head-to-head comparison between traditional resistance training (TRT) and flywheel resistance training (FRT), with standardized exercises, sets, and frequency, is a novel contribution that isolated the specific effects of FRT on cognitive flexibility. The integration of cognitive assessments with serum IGF-1 measurements, although yielding no significant changes, provided valuable insight that the cognitive benefits may arise from central neural mechanisms rather than systemic endocrine pathways. The use of three well-validated neuropsychological tests enabled a multidimensional evaluation of executive functions, revealing domain-specific improvements, particularly the superiority of FRT for cognitive flexibility. Additional strengths include high adherence (only 4 dropouts out of 46), excellent safety (no severe adverse events), pragmatic community-based recruitment enhancing generalizability, and rigorous methodological controls such as blinding of outcome assessors and concealed allocation, all of which strengthen internal validity.

Despite its strengths, this study has several limitations that should be considered when interpreting our findings. The absence of a non-exercising control group limits causal attribution, as we cannot rule out placebo effects, habituation to the testing environment, or spontaneous improvements over time—although the primary aim was to compare two active modalities (FRT vs. TRT) rather than to establish efficacy relative to a no-intervention control. The repeated administration of the same neuropsychological tests at three time points may have introduced practice effects, potentially masking true intervention effects or inflating within-group improvements, despite the relatively long 18-week interval between assessments. Additionally, we did not systematically monitor changes in habitual physical activity, dietary intake, sleep patterns, or other lifestyle factors during the 36-week intervention, which could have independently influenced cognitive outcomes and confounded the specific effects of the training protocols. The exclusive inclusion of community-dwelling older women limits generalizability to men, younger adults, or clinical populations such as those with mild cognitive impairment or dementia, and whether the observed superiority of FRT for cognitive flexibility would be replicated in other groups remains unclear. Other methodological constraints include the relatively small final sample size (46 completers), which reduces statistical power for detecting smaller effects; the absence of formal cognitive screening (e.g., MMSE) to rule out subclinical impairment; the subjective nature of perceived exertion (OMNI-RES) for controlling intensity; the measurement of IGF-1 only, without other neurotrophic factors such as BDNF, limiting mechanistic insights; the lack of neuroimaging or neurophysiological measures to confirm the involvement of specific brain regions; and the absence of long-term follow-up to determine whether the cognitive benefits are sustained after training cessation.

Future research should prioritize larger and more diverse samples, including both sexes, individuals with mild cognitive impairment or at higher risk of cognitive decline (e.g., APOE ε4 carriers, those with metabolic syndrome), and frail older adults, while pragmatic trials with less restrictive criteria and longer follow-ups would assess real-world effectiveness across socioeconomic and cultural backgrounds. To elucidate neurobiological mechanisms, future studies should integrate neurophysiological and neuroimaging techniques such as fMRI (to assess brain activation, connectivity, and structural plasticity in prefrontal and frontoparietal networks), EEG/ERPs (to evaluate temporal dynamics of attention, conflict monitoring, and response inhibition via N200 and P300 components), and NIRS (for prefrontal oxygenation and hemodynamic responses in community settings). Additionally, a broader biomarker panel is recommended beyond IGF-1, including BDNF (synaptic plasticity and neurogenesis), VEGF and IGFBP-3 (angiogenesis and IGF-1 regulation), inflammatory markers (IL-6, TNF-α, CRP), oxidative stress markers, and metabolic parameters, with multi-omics approaches providing comprehensive molecular insights. Finally, future investigations should incorporate long-term follow-up (6–12 months post-intervention) to assess durability of cognitive benefits and dementia risk reduction; include non-exercising control groups to estimate true effect sizes and control for practice effects; use alternative test versions or computerized batteries to minimize learning effects; objectively monitor habitual physical activity and dietary intake to control for confounding lifestyle changes; investigate dose–response relationships to optimize FRT prescription; and conduct translational studies in real-world settings such as community centers, primary healthcare units, and long-term care facilities where access to specialized equipment may be limited.

## 5. Conclusions

This study demonstrates, for the first time in a long-term randomized controlled trial (36 weeks), that flywheel resistance training is superior to traditional resistance training in improving cognitive flexibility in healthy older women. Although both modalities promoted significant improvements in inhibitory control and working memory, FRT was particularly effective in adapting to new cognitive and motor demands, probably due to its greater requirements for motor control, attention, and coordination during the eccentric phase of movement. The absence of significant changes in serum IGF-1 concentrations suggests that the observed cognitive benefits may not be mediated by systemic endocrine pathways involving circulating IGF-1. However, this finding does not exclude the possibility of local central nervous system adaptations, changes in IGF-1 bioavailability, or alterations in other neurotrophic factors (e.g., BDNF, VEGF) not assessed in this study. Future studies incorporating a broader panel of biomarkers and neurophysiological measures are needed to fully elucidate the mechanisms underlying the cognitive benefits of resistance training. These findings have important practical implications for exercise prescription in older adults, positioning FRT as a promising and safe non-pharmacological strategy to mitigate age-related cognitive decline. Future studies with larger samples and including populations with mild cognitive impairment are needed to confirm and expand these findings.

## Figures and Tables

**Table 1:** Summary of inclusion and exclusion criteria for study participation.

Inclusion Criteria	Exclusion Criteria
Female sex	Contraindications to physical exercise (e.g., uncontrolled cardiovascular disease, severe musculoskeletal disorders)
Age ≥ 60 years	Neurological diseases (e.g., Alzheimer’s disease, Parkinson’s disease, stroke, dementia)
Literate (able to read and understand test instructions)	Participation in resistance training programs in the previous three months
Normal or corrected-to-normal vision	Regular physical activity ≥ 150 min/week of moderate-to-vigorous intensity
<150 min/week of moderate-to-vigorous physical activity	Inability to provide informed consent
Voluntary agreement to participate and signed informed consent	Unavailability for the full 36-week intervention period

**Table 2:** Pre-intervention sample characteristics.

Variable	TRT (n = 21)	FWT (n = 25)	*p*	CI 95%
Age (years)	65.5 ± 4.1	68.0 ± 4.8	0.065	−0.16; 5.21
Resting HR (bpm)	67.9 ± 8.0	70.8± 10.6	0.311	−2.80; 8.59
SBP (mmHg)	117.4 ± 8.0	121.4 ± 13.7	0.242	−2.81; 10.84
DBP (mmHg)	79.0 ± 6.2	81.6 ± 8.0	0.241	−1.78; 6.88
MAP (mmHg)	91.7 ± 6.1	94.8 ± 8.6	0.17	−1.38; 7.61
Waist circumference (cm)	89.1 ± 11.1	100.3 ± 11.5	0.002 *	4.47; 18.02
Body mass (kg)	64.1 ± 10.2	74.3 ± 11.8	0.003 *	−0.02; 0.05
BMI (kg/m^2^)	26.4 ± 4.3	29.9 ± 4.7	0.011 *	3.63; 16.90
Number of NCDs	1.0 ± 0.8	1.5 ± 0.9	0.04 *	0.86; 6.27

Data are presented as mean ± standard deviation (M ± SD) and as absolute and relative frequencies. Abbreviations: TRT—traditional resistance training; FRT—flywheel resistance training; HR—heart rate; bpm—beats per minute; SBP—systolic blood pressure; DBP—diastolic blood pressure; MAP—mean arterial pressure; mmHg—millimeters of mercury; cm—centimeters; kg—kilograms; BMI—body mass index; kg/m^2^—kilograms per square meter; NCD—noncommunicable chronic disease. * Statistical significance at *p* < 0.05.

**Table 3:** Effects of the interventions on executive function variables and biomarkers in older women.

	TFWT (n = 25)			TRT (n = 21)			Time			Group		
Variable	Pre	During	Post	Pre	During	Post	*p*	ηp^2^	1 − β	*p*	ηp^2^	1 − β
Vitória Stroop Teste (s)	15.21 ± 10.43	11.72 ± 9.91 *^a^	14.80 ± 7.35 ^a^	20.37± 11.76	17.97 ± 9.61 *^a^	15.6 ± 7.3 ^a^	0.028	0.38	0.686	0.015 *	0.230	0.754
Span de dígitos (direto)	6.24 ± 1.70	7.24 ± 1.61 ^a^	7.90 ± 1.97 ^ab^	6.67 ± 1.49	7.81 ± 2.11 ^a^	8.71 ± 1.8 ^ab^	<0.001	0.844	1.000	0.95	0.00	0.05
Span de dígitos (indireto)	6.29 ± 1.68	6.00 ± 1.55	5.62 ± 1.66	6.43 ± 1.50	5.57 ± 1.40	5.86 ± 1.7	0.505	0.087	0.149	0.852	0.002	0.054
Trail Making Test A (s)	78.35 ± 66.23	73.51± 62.91 ^a^	69.91 ± 48.41 ^a^	70.41± 3.36	54.64 ± 22.02	50.2 ± 21.4	0.007	0.486	0.869	0.242	0.085	0.208
Trail Making Test B (s)	151.4 ± 91.4	155.2 ± 78.8	148.3 ± 93.3	137.7 ± 74.0	142.4 ± 89.9	137.7 ± 83.6	0.561	0.074	0.132	0.719	0.008	0.064
Trail Making Test BA (s)	73.03 ± 65.16	81.71 ± 56.07	87.46 ± 68.06 ^a^	67.34 ± 54.13	87.78 ± 71.88 *	137.7 ± 83.6 ^a^*	0.839	0.011	0.075	0.001 *	0.013	0.072
IGF-1 (ng/mL)	112.90 ± 25.36	-	115.3 ± 21.33	104.7 ± 27.5	-	102.95 ± 28.86	0.88	0.002	0.05	0.5	0.032	0.11

^a^ Significant time effect; ^b^ Significant difference between pre- and post-intervention; * Significant time × group interaction.

## Data Availability

The data that support the findings of this study are available from the corresponding author (O.C.M.) upon reasonable request.
